# Association Between Health-Related Quality of Life and Ultrasound-Based Transverse Rectal Diameter in Pediatric Functional Constipation

**DOI:** 10.3390/children13101316

**Published:** 2026-09-27

**Authors:** Pola Waissman, Shimon Reif, Yaniv Nudelman, Michael Wilschanski, Shmuel Springer, Mordechai Slae

**Affiliations:** 1 Neuromuscular and Human Performance Laboratory, Department of Physiotherapy, Ariel University, Ariel 4070000, Israel; 2 Department of Pediatrics, Hadassah—Hebrew University Medical Center, Jerusalem 9112001, Israel; 3 Department of Physical Therapy, Maccabi Healthcare Services, Tel-Aviv 6812509, Israel

**Keywords:** pediatric functional constipation, transverse rectal diameter, health-related quality of life, pocket-sized ultrasound

## Abstract

**Highlights:**

**What are the main findings?**

- In children with pediatric functional constipation (PFC), larger transverse rectal diameter (TRD) measured using pocket-sized ultrasound (PsUS) was strongly associated with greater constipation severity.
- Larger TRD was also associated with lower parent-reported PedsQL Generic Core, Gastrointestinal Symptoms, and Family Impact scores.

**What are the implications of the main findings?**

- PsUS-derived TRD may complement symptom-based assessment by providing objective information on rectal distension.
- Considering TRD alongside child health-related quality of life and family impact may support a more comprehensive, child- and family-centered evaluation of PFC.

**Abstract:**

Background: Pediatric functional constipation (PFC) significantly impairs children’s daily functioning and health-related quality of life (HRQoL). Assessing baseline severity and ensuring treatment progression are key to successful management. Pocket-sized ultrasound (PsUS) offers an accessible, dynamic, quantitative assessment of rectal morphology, but it is unclear whether PsUS-derived transverse rectal diameter (TRD) may reflect patient-relevant functional burden. This study examined the associations between PsUS-derived TRD, constipation severity, and parent-reported HRQoL in children with PFC. Methods: This cross-sectional study included 47 children (mean age 7.22 ± 2.04 years; 62% boys) with Rome IV-defined PFC. TRD was measured using PsUS, constipation severity using the Wexner Constipation Scoring System (WCSS), and parent-reported outcomes using the PedsQL™ Generic Core Scales, Gastrointestinal Symptoms Module, and Family Impact Module. Associations were analyzed using Spearman correlations. Results: The cohort had a mean age of 7.22 ± 2.04 years. Mean PsUS-derived TRD was 36.5 ± 6.6 mm, consistent with rectal diameters reported in children with functional constipation. Mean constipation severity (WCSS) was 21.5 ± 2.8 (0–30 scale; higher scores indicating greater severity), and mean PedsQL scores were 28.7 ± 17.1 for the Gastrointestinal Symptoms Module, 21.8 ± 16.7 for the Family Impact Module, and 21.1 ± 16.7 for the Generic Core Scale (0–100 scale, higher indicating better HRQoL). Larger TRD values were strongly associated with poorer HRQoL across the PedsQL™ 4.0 Generic Core Scales, Gastrointestinal Symptoms Module, and Family Impact Module (ρ = −0.67 to −0.87, all *p* < 0.001). TRD also showed a very strong positive association with constipation severity (WCSS; ρ = 0.91, *p* < 0.001). Conclusions: Larger PsUS-derived TRD values were associated with greater constipation severity and poorer parent-reported HRQoL in children with PFC. These findings suggest that PsUS-derived TRD may provide objective information related to both constipation severity and parent-reported quality-of-life outcomes.

## 1. Introduction

Pediatric functional constipation (PFC) is a major gastrointestinal disorder affecting up to 14% of children worldwide [1,2], making it one of the most common conditions in pediatric gastroenterology [3]. Its etiology is multifactorial, involving physiological, psychological, social, cultural, and behavioral factors, which together present significant challenges for diagnosis and management [3,4,5]. Beyond intestinal dysfunction, PFC significantly impacts health-related quality of life (HRQoL), as affected children often face limitations in physical, social, and academic areas [6]. Families frequently report increased stress and caregiving demands, and the disorder is associated with higher healthcare utilization and school absenteeism [7,8].

The evaluation of PFC is currently based on the Rome IV criteria, which are widely endorsed by the North American and European Societies of Pediatric Gastroenterology, Hepatology, and Nutrition (NASPGHAN and ESPGHAN) [9]. Although the Rome IV criteria provide a standardized symptom-based definition of functional constipation, they do not assess disease severity or directly evaluate rectal morphology and physiology, factors that may contribute to a more comprehensive understanding of the clinical context underlying symptom presentation [10]. This has increased interest in quantifiable measures that can complement symptom-based assessment and support a more comprehensive clinical characterization of PFC. One such measure is transabdominal ultrasonography (US), a reproducible, validated, non-invasive, and child-friendly technique for assessing rectal morphology in children, allowing repeated examinations without radiation exposure [10,11]. This imaging modality enables visualization of rectal contents and wall structure, and can effectively detect conditions such as fecal impaction and megarectum (pathological rectal dilatation due to chronic stool retention), potentially reducing the need for digital rectal examinations in clinical practice [10,12,13,14]. Transverse rectal diameter (TRD) and rectal anterior wall thickness (RAWT) have been proposed as two suitable sonographic parameters for assessing fecal retention and rectal adaptation in PFC [15,16]. TRD has been more extensively studied, with evidence consistently demonstrating larger diameters and good discriminatory performance in children with PFC compared with healthy controls. In contrast, the evidence supporting RAWT as a marker of chronic rectal remodeling is less established, limited, and heterogeneous [10,11,12,13,14,15,16,17,18,19,20,21].

While quantifiable physiological measures are important in assessing PFC, evaluating HRQoL provides additional insight into the broader functional and psychosocial impact of the disorder from the perspective of children and their families [22]. Impaired HRQoL has been consistently associated with greater pain, fecal incontinence, and overall constipation severity in pediatric functional gastrointestinal disorders (FGIDs) [6,7,23,24,25]. HRQoL is increasingly recognized as a key outcome in pediatric constipation research and practice, and is typically assessed using standardized questionnaires completed by children and/or their parents, depending on the child’s age and study design [6,26]. Among these instruments, the Pediatric Quality of Life Inventory (PedsQL) is widely used and has demonstrated clinical utility in this population [17,27,28], capturing patient-relevant outcomes beyond symptoms and imaging [6,8].

Although both US-based rectal measurement and HRQoL impairment are well documented in PFC, they represent fundamentally different parameters of the disorder. Ultrasonography provides quantitative information on rectal morphology and physiological loading, whereas HRQoL captures the child’s and family’s lived functional and psychosocial burden. Consistent with biopsychosocial frameworks [29], these domains are complementary and are both needed for comprehensive assessment of PFC [10,30,31]. However, it remains unclear whether US-assessed rectal morphology meaningfully corresponds with the functional impact experienced by children and families. Clarifying this relationship is clinically relevant because it may indicate whether sonographic parameters can serve as adjunct markers of disease burden that complement symptom-based evaluation and patient-reported outcomes. Such correspondence may help clinicians interpret patient- and family-reported burden in relation to measurable rectal morphology, thereby strengthening the clinical context of symptom-based assessment. Accordingly, this exploratory study examined the associations between TRD, constipation symptom severity, and HRQoL in children with PFC, to characterize the direction and magnitude of these relationships.

## 2. Materials and Methods

### 2.1. Study Design and Participants

This cross-sectional study included children aged 4–14 years diagnosed with PFC. This age range was selected to include a clinically coherent cohort of children and early adolescents with pediatric functional constipation. The lower limit reflects the Rome IV distinction for children with a developmental age of at least 4 years, whereas the upper limit was chosen to reduce developmental and psychosocial heterogeneity associated with later adolescence. Participants were recruited between May 2024 and July 2025 from the Pediatric Gastroenterology Outpatient Clinic at Hadassah Medical Center, after referral for evaluation of constipation-related complaints, with recruitment supported by nationwide outreach. Recruitment occurred during routine outpatient clinic visits after study approval. All included children had constipation-related symptoms for at least one year, had received conservative management before enrollment, and were receiving polyethylene glycol (PEG) at the study assessment. Eligibility was confirmed by a pediatric gastroenterologist according to the Rome IV criteria at the time of the study assessment. At the study assessment, ongoing constipation-related burden was further characterized by constipation severity and rectal distension, assessed using the Wexner Constipation Scoring System and pocket-sized ultrasound-derived TRD, respectively. Children were excluded if they had congenital malformations of the digestive tract (such as Hirschsprung’s disease or anorectal malformations), anatomical rectal anomalies, neurological disorders affecting bowel function (such as spina bifida or cerebral palsy), prior gastrointestinal surgery, chronic systemic or metabolic diseases, severe psychiatric or behavioral conditions that could interfere with study participation, or open wounds near the symphysis pubis that precluded US measurement. Each child underwent PsUS-based measurement of TRD, along with parent-reported assessments of HRQoL and constipation severity. The study was approved by the institutional ethics committee of Hadassah—Hebrew University Medical Center (Protocol No. HMO-0446-23). Written informed consent was obtained from the legal guardians of all participants. Participation was voluntary, and families could withdraw at any time.

### 2.2. Data Collection

Each child attended a standardized 30-min study visit during which parent-reported questionnaires assessing HRQoL and constipation severity were completed, and TRD was measured using transabdominal ultrasonography with PsUS. Anthropometric data were collected using standard procedures, with BMI calculated from measured height and weight. To facilitate interpretation across the broad pediatric age range, weight, height, and BMI were presented as age- and sex-adjusted percentiles calculated using CDC 2000 growth reference charts. Weekly bowel habit and symptom-related data were prospectively recorded by parents using a study-specific weekly stool diary (Supplementary File S1). These data included stool and defecation frequency, weekly fecal leakage frequency, blood in stool, weekly stool volume, parent-reported current PEG use and usual daily dose, and use of other medications. Stool consistency was assessed using the Bristol Stool Form Scale (1–7) [32], and defecation-related symptoms (pain, effort, urgency) were rated on visual analogue scales (VAS; 0–10) [33] as average scores over the preceding week, with higher scores indicating greater defecation difficulty.

#### 2.2.1. Ultrasound-Based TRD Assessment

TRD measurements were performed by a pediatric pelvic floor clinician with 15 years of clinical experience, trained according to the Constipation POCUS Educational Program developed by Matsumoto et al. [34]. Matsumoto et al. describe the Constipation POCUS Educational Program as a structured educational program that can be completed in as little as 7 days. The program includes 2 h of e-learning, a 5-h hands-on seminar, self-learning within 1 week, and 30-min objective structured clinical examinations (OSCEs). Measurements were obtained using a pocket-sized ultrasound device (PsUS; Kosmos ultrasound system, EchoNous Inc., Redmond, WA, USA) equipped with a 2–5 MHz convex probe. Each examination lasted approximately five minutes and was performed transabdominally using an external abdominal probe, without rectal instrumentation or other invasive procedures. During the examination, the child was positioned supine, with the head elevated by 10° and the knees slightly flexed. Scans were performed with a full bladder to optimize visualization. No participant underwent an enema, disimpaction, or other induced stool evacuation during the month preceding the US examination. The probe was positioned 2 cm above and parallel to the pubic symphysis and angled 10–15° caudally. TRD was measured in the transverse plane as the maximum distance between the lateral rectal walls. The widest section of the rectal ampulla was identified across multiple frames, and a single TRD value was recorded from the selected frame using a standardized cursor placement protocol to ensure consistency. The sonographer was blinded to the participants’ WCSS and PedsQL responses and scores during image acquisition and measurement. US images were included for analysis only when the bladder was adequately filled to provide a clear acoustic window and when rectal wall margins were distinctly delineated. TRD was analyzed as a continuous variable, with larger values reflecting greater rectal distension [12,15,16,35].

#### 2.2.2. Health-Related Quality of Life

Health-related quality of life outcomes were assessed using three validated Pediatric Quality of Life Inventory (PedsQL™) instruments: the PedsQL 4.0 Generic Core Scales, the PedsQL Gastrointestinal Symptoms Module, and the PedsQL Family Impact Module. All PedsQL forms were completed by parents. The PedsQL 4.0 Generic Core Scales and Gastrointestinal Symptoms Module were completed using the age-appropriate parent-proxy versions according to each child’s age at the study assessment; the PedsQL Family Impact Module was completed using its standard parent-report format, which is not stratified by child age. These instruments have demonstrated good reliability, validity, and responsiveness in pediatric populations, including children with functional gastrointestinal disorders [23,32,33,36]. All items are rated on a 5-point Likert scale, reverse-scored, and transformed to a 0–100 scale, with higher scores indicating better HRQoL. The Generic Core Scales provide an overall HRQoL score across physical, emotional, social, and school functioning domains. The Gastrointestinal Symptoms Module yields a gastrointestinal-specific HRQoL score, and the Family Impact Module assesses the impact of the child’s condition on parent HRQoL and family functioning [32,33,37,38].

#### 2.2.3. Constipation Severity

Constipation symptom severity was assessed using the Wexner Constipation Scoring System (WCSS), also known as the Cleveland Clinic Constipation Score (CCCS) [39]. The WCSS comprises eight items evaluating defecatory function, including bowel movement frequency, difficulty with defecation, incomplete evacuation, abdominal pain, time spent on the toilet, need for assistance, unsuccessful attempts, and duration of constipation. Each item is scored on an ordinal scale, yielding a total score ranging from 0 to 30, with higher scores indicating greater symptom severity. The WCSS was originally developed and validated in adults [39] and has subsequently been used or adapted in pediatric studies involving functional constipation and other bowel disorders [40,41,42,43,44,45]. In the present study, the original eight-item WCSS was completed by a parent; no formally validated pediatric version was used.

### 2.3. Statistical Analysis

A priori power analysis for a two-tailed correlation test (α = 0.05), conducted using G*Power 3.1 [46], indicated that a sample size of 46 participants would provide 80% power to detect a moderate association between TRD and HRQoL, as assessed across the PedsQL domains described above. Descriptive statistics are reported as mean ± standard deviation and median (interquartile range) for demographic and outcome measures. Variable distributions were examined using histograms and Q-Q plots. Potential outliers, defined as values exceeding 2 × IQR below the first quartile or above the third quartile, were identified; values were retained unless clinical or measurement-based evidence indicated implausibility. One WCSS value (12/30) met this criterion but was retained, representing a plausible instance of milder symptom severity. The seven primary correlations were repeated after excluding this participant as a sensitivity analysis (Appendix C). Bivariate associations between TRD, PedsQL scores, and constipation severity (WCSS) were examined using Spearman rank correlation coefficients (ρ), based on observed non-normal distributions. Associations between WCSS and PedsQL were additionally examined to assess consistency with previously reported patterns. Correlation strength was interpreted as negligible (<0.20), weak (0.20–0.39), moderate (0.40–0.59), strong (0.60–0.79), or very strong (≥0.80) [47], with Bonferroni adjustment applied to prespecified tests (TRD with PedsQL domains and WCSS, and WCSS with PedsQL domains) to control Type I error [48]. Additional Spearman correlations were conducted to examine associations between TRD and key clinical and demographic characteristics. These analyses were considered secondary, and Bonferroni correction was applied across the 14 correlations, yielding an adjusted significance threshold of *p* < 0.0036. To examine adjusted associations, multivariable regression models were fitted, controlling for age, sex, BMI, and daily PEG dose. Linear and second-degree polynomial terms were evaluated when scatterplots suggested non-linearity. Association magnitude was quantified using standardized regression coefficients (β), with values of 0.1, 0.3, and 0.5 indicating small, medium, and large effects, respectively [49]. Analyses were conducted using R 4.6.0, (RStudio) [50,51], with data management performed using the “tidyverse” package (version 2.0.0) [52].

As a complementary approach to the covariate-adjusted regression models described above, a sensitivity analysis was conducted to assess the consistency of the primary associations across age groups. This was motivated by the broad pediatric age range of the cohort (4–14 years) and the absence of standardized, age-adjusted normative TRD reference values for children in this range. Age tertiles were defined using the empirical 33.3rd and 66.7th percentiles of age, yielding groups of 16, 15, and 16 participants. The primary Spearman correlations between TRD and constipation severity (WCSS) and HRQoL domains were re-examined within each tertile (Appendix B).

## 3. Results

### 3.1. Participant Characteristics

A total of 50 children were recruited, of whom 47 were included in the final analysis. Two participants were excluded due to technically inadequate TRD images, and one withdrew prior to completing study procedures. The final analytical sample comprised 47 children (mean age 7.22 ± 2.04 years, 62% boys). Descriptive statistics for all study variables are presented in Table 1.

### 3.2. Associations Between PsUS-Based Rectal Diameter and HRQoL

PsUS-derived TRD showed strong to very strong associations with constipation severity and all assessed HRQoL domains (Table 2, Figure 1). TRD was inversely correlated with the PedsQL Generic Core Scale (ρ = −0.87, *p* < 0.001), the Gastrointestinal Symptoms Module (ρ = −0.72, *p* < 0.001), and the Family Impact Module (ρ = −0.67, *p* < 0.001), and was very strongly correlated with constipation severity as measured by the WCSS (ρ = 0.91, *p* < 0.001).

Multivariable regression analyses confirmed the magnitude and direction of these associations after adjustment for age, sex, BMI, and daily PEG dose (all *p* < 0.001). Standardized regression coefficients were −0.75 for the Generic Core Scale, −0.71 for the Gastrointestinal Symptoms Module, −0.69 for the Family Impact Module, and 0.81 for constipation severity. Covariates generally demonstrated negligible, non-significant associations with constipation severity and HRQoL, with the exception of the Family Impact Module: BMI (β = −0.33, *p* = 0.004) and daily PEG dosage (β = 0.24, *p* = 0.031) were both significantly associated with Family Impact scores, independent of TRD.

For the PedsQL Generic Core Scale, a second-degree polynomial model provided a significantly better fit than the linear model (F(1,40) = 15.76, *p* < 0.001). Both the linear and quadratic TRD terms were statistically significant (*p* < 0.001). No significant non-linear effects were observed for other outcomes.

### 3.3. Associations Between Constipation Severity and HRQoL

Constipation severity was also strongly and inversely associated with HRQoL across all domains, including the Generic Core Scale (ρ = −0.82), Gastrointestinal Symptoms Module (ρ = −0.74), and Family Impact Module (ρ = −0.69), all *p* < 0.001. Sensitivity analysis excluding the participant with a WCSS score of 12 yielded similar primary correlation estimates, with absolute differences in ρ below 0.05. All seven correlations remained significant after Bonferroni correction (Appendix C).

### 3.4. Associations Between PsUS-Based Rectal Diameter and Clinical and Demographic Characteristics

TRD was moderately positively correlated with total weekly fecal leakage (events/week; ρ = 0.52, *p* < 0.001) and moderately negatively correlated with weekly stool volume (teaspoons/week; ρ = −0.40, *p* = 0.005). The association with fecal leakage remained statistically significant after Bonferroni correction for 14 comparisons (adjusted significance threshold, *p* < 0.0036); the association with stool volume did not survive correction at this threshold. No other associations met the corrected significance threshold.

### 3.5. Sensitivity Analysis: Stratification by Age

To assess whether the primary TRD associations were consistent across age-defined subgroups, we performed a sensitivity analysis stratified by age into tertiles (*n* = 15–16 per subgroup). Descriptive characteristics of each age tertile are presented in Appendix A, and the corresponding correlations in Appendix B. Across age tertiles, the associations remained statistically significant and were strong to very strong in magnitude for HRQoL and very strong for constipation severity, consistent with the non-stratified findings (Appendix B). Specifically, correlations between TRD and constipation severity ranged from 0.90 to 0.93 (all *p* < 0.001), and correlations between TRD and HRQoL domains ranged from −0.65 to −0.90 (all *p* ≤ 0.008). The subgroup estimates were consistent in direction with the whole-cohort findings and were interpreted as exploratory given the small subgroup sizes.

## 4. Discussion

Functional constipation in children is a complex condition involving physiological disturbances, alongside significant emotional, social, and functional challenges for children and their families [20,54,55]. However, the extent to which physiological measures correspond with functional and psychological symptom burden remains incompletely characterized [9,56]. This exploratory study examined whether PsUS-derived TRD is associated with constipation symptom severity and HRQoL in children with PFC. To our knowledge, this is the first study to evaluate these relationships within the same cohort. We found that larger TRD values were associated with greater constipation severity and poorer HRQoL across all assessed PedsQL domains, and these associations remained significant after adjustment for age, sex, BMI, and daily PEG dose. In the secondary analyses, larger TRD was also associated with higher weekly fecal leakage frequency after correction for multiple comparisons. Taken together, these findings indicate that PsUS-derived TRD is related to symptom severity and HRQoL in PFC, supporting its relevance as an objective marker of rectal distension.

Our findings are consistent with a recent prospective pediatric study by Proli et al. [57], which reported an association between TRD and constipation severity and demonstrated reductions in TRD within two months of treatment initiation, supporting its potential role in monitoring physiological change. Importantly, that study did not examine quality of life. In this context, our findings extend existing evidence by linking TRD not only to symptom severity but also to HRQoL within the same cohort. From a pathophysiological perspective, this relationship is biologically plausible, as chronic stool retention in pediatric functional constipation contributes to progressive rectal dilatation and reduced rectal sensation, sustaining a self-perpetuating cycle in which greater rectal distension is associated with higher symptom burden [58].

The PedsQL is a validated tool for assessing HRQoL in pediatric gastrointestinal conditions, including functional constipation [24,59,60,61,62]. In the present cohort, mean PedsQL scores were 21.1 for the Generic Core Scale, 28.7 for the Gastrointestinal Symptoms Module, and 21.8 for the Family Impact Module. By comparison, a meta-analysis reported a pooled total HRQoL score of 65.6 in children with functional constipation versus 86.1 in healthy controls, with lower scores in hospital-based than community-based cohorts [6]. Although these estimates are not directly comparable because of differences in populations and HRQoL measures, they provide useful context for the consistently low scores observed in our cohort and the substantial burden experienced by children and families. The prolonged symptom duration, prior conservative management, ongoing PEG use, and specialist referral setting indicate that participants represented a treatment-experienced population with persistent PFC. This clinical context should be considered when interpreting the magnitude of the observed associations.

Across many chronic conditions, objective physiological or structural markers often show weak or inconsistent correspondence with symptom burden and functional impact, such as lumbar spine degenerative changes [63], juvenile idiopathic arthritis [64], or pediatric inflammatory bowel disease [65]. In contrast, we observed a strong alignment between PsUS-derived TRD and HRQoL in children with PFC. This pattern may further indicate that TRD reflects a disease-relevant physiological state in PFC that is closely linked to functional impact.

TRD was most strongly associated with the PedsQL Generic Core Scale, with a non-linear relationship suggesting that differences in rectal diameter may not correspond to uniform differences in overall HRQoL across the observed range. The strong associations between TRD, gastrointestinal symptom-specific HRQoL, and family impact suggest that rectal distension relates not only to child-level symptoms but also to broader family functioning. In the adjusted linear model, higher BMI was associated with lower Family Impact scores, whereas a higher daily PEG dose was associated with higher scores. TRD remained strongly associated with Family Impact after adjustment for these covariates. From a clinical perspective, the observed association between larger PsUS-derived TRD, greater symptom severity, and poorer HRQoL suggests that rectal distension may provide information that complements symptom-based assessment in children with PFC. In clinical practice, identifying substantial rectal distension may encourage a more comprehensive evaluation of symptom burden and its impact on the child and family, and may prompt consideration of treatment reassessment. Beyond symptom severity alone, management of PFC should consider the child’s and family’s HRQoL. In practice, greater awareness of the potential relationship between rectal distension, symptom burden, and functional impact may help clinicians and families better understand the condition and support shared decision-making. Treatment of PFC may need to extend beyond bowel symptom optimization and may encompass targeted family education, parental counseling, and behavioral or psychosocial support, guided by the child’s clinical presentation and family needs [55,66,67]. For families specifically, explaining the clinical significance of rectal distension, including its contribution to fecal leakage and the chronic nature of rectal remodeling, may help reduce parental blame, foster a more accurate understanding of the child’s difficulties, and strengthen commitment to home management [30,59]. The noninvasive and repeatable nature of TRD assessment makes it potentially suitable for longitudinal evaluation [68]. However, the present cross-sectional study did not evaluate within-participant changes in TRD or responsiveness to treatment. Prospective studies are needed to determine whether changes in TRD correspond to clinical improvement and can inform patient follow-up.

Several limitations should be considered when interpreting these findings. The cross-sectional design precludes conclusions regarding temporal relationships or responsiveness to treatment. Although the sample size was sufficient to detect the full-cohort associations, larger cohorts are needed for more precise subgroup and longitudinal analyses. Prospective follow-up is needed to determine whether changes in PsUS-derived TRD correspond to changes in symptom severity and HRQoL over time. The WCSS was originally developed and validated in adults, and the parent-completed version used in this study has not undergone formal psychometric validation in children with PFC. Although the instrument has previously been used or adapted in pediatric research, this limitation should be considered when interpreting WCSS scores and the magnitude of their associations with TRD and HRQoL. The selected clinical profile and specialist clinic setting may have influenced the magnitude of the observed associations and may limit their generalizability to children with shorter symptom duration or those managed in community settings. Furthermore, HRQoL findings were based on parent-reported PedsQL data, which may not fully capture the child’s perspective, although parent-reported assessment is widely accepted in pediatric research. Interpretation of TRD should also account for the absence of observer reliability data, the unrecorded timing of the last spontaneous bowel movement, and potential age- and body-size-related variation across the 4–14 year cohort. To date, no validated age- or body-size-adjusted TRD reference standards or pediatric percentile curves are available and published diagnostic cut-off values for functional constipation vary widely across studies (approximately 24–38 mm) [10]. Accordingly, absolute TRD values may vary with physical growth and cannot yet be interpreted uniformly across children of different ages and body sizes. In the absence of an established age- or body-size-standardized measure, absolute TRD was retained as the primary ultrasound measure. Multivariable models were adjusted for age, sex, BMI, and daily PEG dose, and exploratory age-stratified sensitivity analyses showed consistent associations across subgroups (Appendix B). Future studies should establish normative TRD percentile curves adjusted for age, sex, and body size to enable standardized pediatric comparisons.

## 5. Conclusions

This exploratory study shows that PsUS-derived TRD is associated with constipation severity and parent-reported HRQoL outcomes in children with PFC. These findings suggest that TRD may serve as a useful adjunct to symptom-based assessment, as larger TRD values were associated with greater child and family burden captured through caregiver-reported outcomes. Larger longitudinal studies are needed to confirm these associations and determine whether repeated TRD assessment can support clinical assessment, family-centered care, and monitoring of clinical change over time.

## Figures and Tables

**Figure 1:**
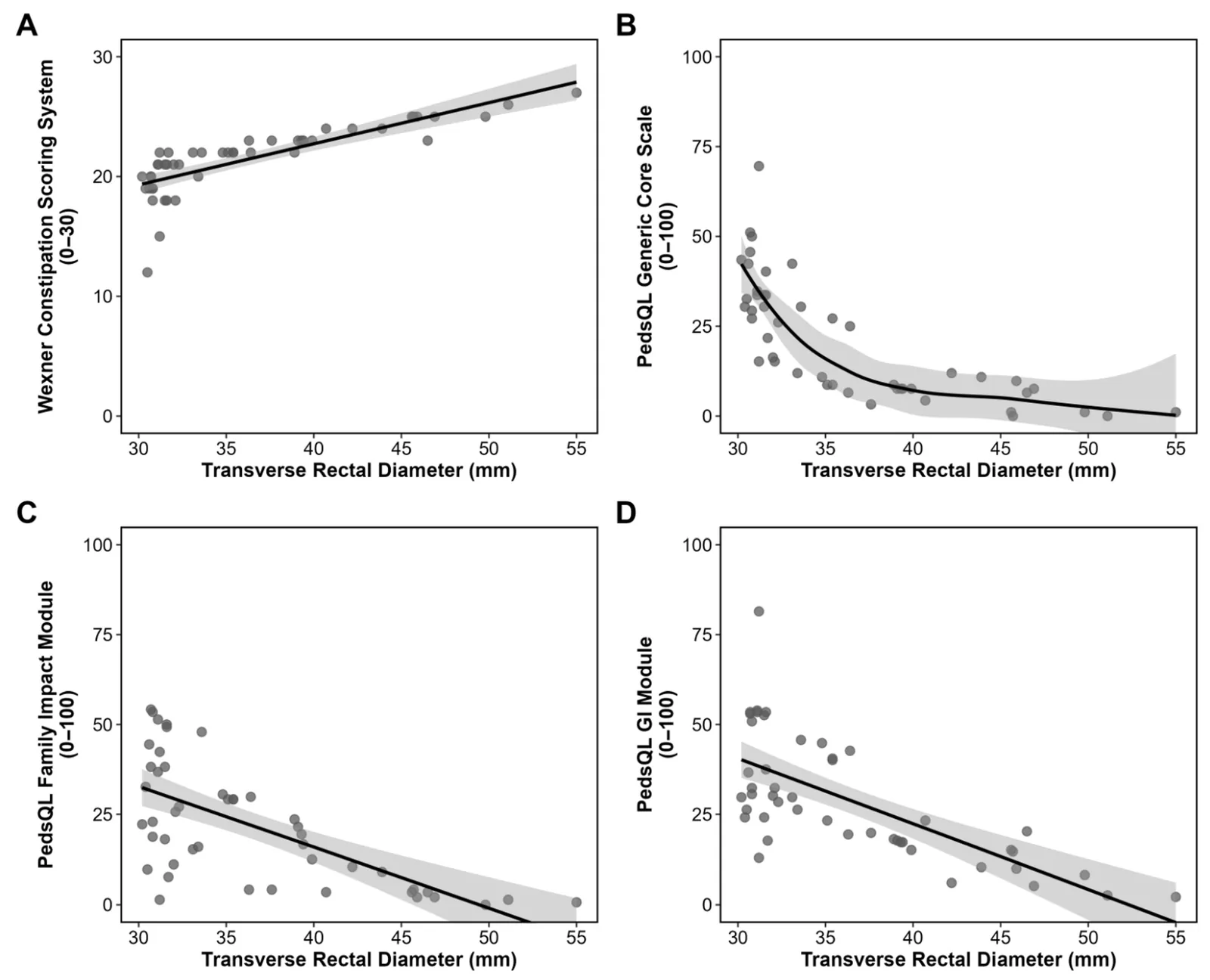
Scatterplots of associations between transverse rectal diameter, symptom severity, and health-related quality of life indices. Associations between transverse rectal diameter (TRD) and clinical outcomes in children with PFC (*n* = 47). Panel (**A**) shows a positive association between TRD and Wexner Constipation Scoring System. Panels (**B**–**D**) show inverse associations between TRD and Pediatric Quality of Life Inventory (PedsQL) domains (Generic Core Scale, Family Impact Module and Gastrointestinal Symptoms Module). Solid lines represent fitted regression functions (linear models for Panels (**A**,**C**,**D**); quadratic regression model for Panel (**B**)), with shaded 95% confidence intervals. Higher PedsQL scores indicate better health-related quality of life, whereas higher Wexner Constipation Scoring System scores indicate greater symptom severity.

**Table 1:** Participant characteristics and descriptive statistics of the study cohort (*n* = 47).

Variable			Mean ± SD	Median (IQR)
Participants characteristics	Age (years)		7.22 ± 2.04	7.07 (3.34)
	Sex	Male, *n* (%)	29 (61.7)	-
		Female, *n* (%)	18 (38.3)	
	Weight percentile		44.32 ± 30.41	40.11 (50.85)
	Height percentile		39.05 ± 33.05	36.85 (47.57)
	BMI percentile		49.31 ± 35.18	48.37 (69.47)
Bowel habits and stool characteristics	PEG (current dose g/day)		14.11 ± 5.67	12 (6)
	Weekly stool volume (teaspoons/week)		6.23 ± 4.51	6 (5.5)
	Blood in stool (events/week)		0.43 ± 1.17	0 (0)
	Total fecal leakage per week (events/week)		9.72 ± 11.49	7 (8)
	Spontaneous bowel movements (events/week)		3.30 ± 2.63	3 (2)
	Mean weekly Bristol stool form (1–7)		3.60 ± 1.99	3 (3.5)
	Pain during defecation (weekly average using VAS 0–10)		7.60 ± 2.82	9 (1.5)
	Defecation effort (weekly average using VAS 0–10)		7.81 ± 2.43	9 (2)
	Urgency during defecation (weekly average using VAS 0–10)		6.85 ± 2.40	7 (3)
Quality of Life (PedsQL)	Gastrointestinal Symptoms Module (0–100)		28.73 ± 17.10	26.29 (23.06)
	Family Impact Module (0–100)		21.81 ± 16.66	19.44 (25.69)
	Generic Core Scale (0–100)		21.09 ± 16.67	15.22 (25.54)
Rectal measurement—TRD (mm)			36.50 ± 6.57	33.60 (8.45)
Constipation severity (WCSS score 0–30)			21.53 ± 2.82	22 (3)

Values are presented as mean ± standard deviation and median (IQR; interquartile range), as appropriate; categorical variables are presented as *n* (%). Weight, height, and BMI percentiles were calculated using CDC growth reference charts adjusted for age and sex [53]. BMI, Body Mass Index; PEG, polyethylene glycol; PedsQL, Pediatric Quality of Life Inventory; TRD, transverse rectal diameter; VAS, Visual Analogue Scale; WCSS, Wexner Constipation Scoring System.

**Table 2:** Associations between ultrasonographic rectal measurement, constipation severity, and health-related quality of life in pediatric functional constipation.

Variable 1	Variable 2	Spearman’s ρ	Standardized β	*p* Value
TRD	PedsQL Generic Core Scale	−0.87	−0.75	<0.001
	PedsQL Gastrointestinal Symptoms Module	−0.72	−0.71	<0.001
	PedsQL Family Impact Module	−0.67	−0.69	<0.001
	WCSS	0.91	0.81	<0.001
WCSS	PedsQL Generic Core Scale	−0.82	-	<0.001
	PedsQL Gastrointestinal Symptoms Module	−0.74	-	<0.001
	PedsQL Family Impact Module	−0.69	-	<0.001

Standardized β coefficients are reported for TRD models only and were obtained from multivariable linear models adjusted for age, sex, BMI, and daily PEG dose (g/day). Standardized β, standardized regression coefficient; ρ, Spearman’s rank correlation coefficient; TRD, transverse rectal diameter; WCSS, Wexner Constipation Scoring System; PedsQL, Pediatric Quality of Life Inventory (Generic Core Scale, Gastrointestinal Symptoms Module, and Family Impact Module); HRQoL, health-related quality of life. Spearman’s ρ and corresponding *p*-values are reported for all bivariate associations. Statistical significance was assessed using a Bonferroni-adjusted threshold of *p* < 0.0071 for the seven prespecified primary correlations.

## Data Availability

The de-identified data supporting the findings of this study are available from the corresponding author upon reasonable request, subject to applicable institutional and ethical requirements. The data are not publicly available due to privacy considerations related to pediatric participants and their families.

## Supplementary Materials

The following supporting information can be downloaded at: https://www.mdpi.com/article/10.3390/children13101316/s1, File S1: Study-specific weekly stool diary.

## Abbreviations

The following abbreviations are used in this manuscript:

FGID	Functional Gastrointestinal Disorder
HRQoL	Health-Related Quality of Life
PEG	Polyethylene Glycol
PedsQL	Pediatric Quality of Life Inventory
PFC	Pediatric Functional Constipation
PsUS	Pocket-Sized Ultrasound
RAWT	Rectal Anterior Wall Thickness
TRD	Transverse Rectal Diameter
US	Ultrasonography
WCSS	Wexner Constipation Scoring System

## Appendix A

**Table A1:** Descriptive characteristics of clinical and quality-of-life outcomes across age tertiles.

Characteristic	Age Tertiles		
	T1	T2	T3
*n*	16	15	16
Age (years)	5.14 ± 0.35 (4.5–5.7)	6.97 ± 0.78 (5.8–8.2)	9.55 ± 1.27 (8.2–13.2)
Height (cm)	112.62 ± 9.11 (100.0–130.0)	118.70 ± 8.51 (101.0–130.0)	127.75 ± 14.72 (103.0–160.0)
TRD (mm)	36.25 ± 6.89 (30.6–55.0)	35.63 ± 5.55 (30.5–46.9)	37.56 ± 7.36 (30.2–51.1)
WCSS (0–30)	21.81 ± 2.20 (18.0–27.0)	20.60 ± 3.72 (12.0–25.0)	22.12 ± 2.31 (18.0–26.0)
PedsQL Generic Core	22.28 ± 14.58 (1.1–45.7)	21.45 ± 20.72 (0.0–69.6)	19.57 ± 15.33 (0.0–43.5)
PedsQL GI Symptoms	33.05 ± 17.16 (2.2–53.9)	29.31 ± 19.24 (5.2–81.5)	23.87 ± 14.53 (2.6–53.4)
PedsQL Family Impact	27.43 ± 15.61 (0.7–50.0)	20.88 ± 17.49 (2.1–54.2)	17.06 ± 16.23 (0.0–51.4)

Values are presented as mean ± standard deviation (minimum-maximum), except n. T1, T2, and T3 represent the youngest, middle, and oldest age tertiles, respectively. PedsQL, Pediatric Quality of Life Inventory; WCSS, Wexner Constipation Scoring System; TRD, transverse rectal diameter.

## Appendix B

**Table A2:** Sensitivity analysis: associations between transverse rectal diameter (TRD), constipation severity, and health-related quality of life across age tertiles.

Stratification	Subgroup (*n*)	PedsQL Generic Core ρ (*p*)	PedsQL GI Symptoms ρ (*p*)	PedsQL Family Impact ρ (*p*)	WCSS ρ (*p*)
Age tertiles	T1 (*n* = 16)	−0.90 (<0.001)	−0.77 (<0.001)	−0.84 (<0.001)	0.93 (<0.001)
	T2 (*n* = 15)	−0.90 (<0.001)	−0.80 (<0.001)	−0.66 (0.008)	0.90 (<0.001)
	T3 (*n* = 16)	−0.86 (<0.001)	−0.65 (0.007)	−0.70 (0.002)	0.91 (<0.001)

Associations were calculated using Spearman correlation coefficients (ρ) between TRD and each outcome within each age tertile; all associations were statistically significant (*p* ≤ 0.008). *p*-values are unadjusted; these subgroup analyses were exploratory. Age tertiles were derived from the age distribution of the study sample (age range: 4–14 years): T1 represents the youngest tertile, T2 the middle tertile, and T3 the oldest tertile. ρ, Spearman’s rank correlation coefficient; PedsQL, Pediatric Quality of Life Inventory; WCSS, Wexner Constipation Scoring System; TRD, transverse rectal diameter; *p*, *p*-value.

## Appendix C

**Table A3:** Sensitivity analysis excluding the participant with a WCSS score of 12.

Association		Primary Analysis, *n* = 47: ρ	Sensitivity Analysis, *n* = 46: ρ
TRD (mm)	WCSS (0–30)	0.907	0.903
	PedsQL Generic Core	−0.866	−0.871
	PedsQL GI Symptoms	−0.719	−0.740
	Family Impact	−0.666	−0.711
WCSS (0–30)	PedsQL Generic Core	−0.823	−0.829
	PedsQL GI Symptoms	−0.738	−0.761
	PedsQL Family Impact	−0.686	−0.735

All correlations had unadjusted *p*-values < 0.001 and remained significant after Bonferroni correction for seven comparisons in both analyses. ρ, Spearman’s rank correlation coefficient; PedsQL, Pediatric Quality of Life Inventory; WCSS, Wexner Constipation Scoring System; TRD, transverse rectal diameter; *p*, *p*-value.
