# Supplementary material for: Association Between Health-Related Quality of Life and Ultrasound-Based Transverse Rectal Diameter in Pediatric Functional Constipation
Source: Children (Basel). 2026 Sep 27;13(10):1316. doi: 10.3390/children13101316 (PMC13703125; doi:10.3390/children13101316)
Supplement: Supplementary file 1 [file children-13-01316-s001.zip › children-4552739-supplementary.pdf]

Bowel Movement Diary

Subject ID:__________

polaw.pt@gmail.com

Date and Time	Stool Type (Bristol Stool Chart) and Estimated Stool Volume (teaspoons) 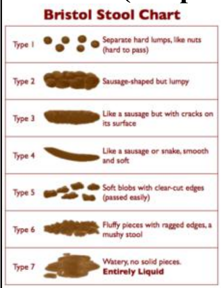	Urgency during Defecation 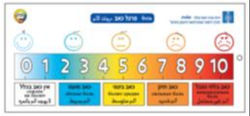	Defecation Effort 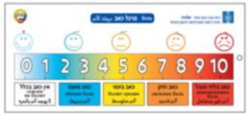	Pain during Defecation 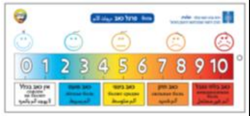	Fecal Leakage Amount (teaspoons)	Abdominal Pain (Yes/No)	Blood in Stool (Yes/No)	Daily PEG Dose / Other Medications	Notes
